# Using Ecological Momentary Assessment to Study the Development of COVID-19 Worries in Sweden: Longitudinal Study

**DOI:** 10.2196/26743

**Published:** 2021-11-29

**Authors:** Peter Johannes Schulz, Elin M Andersson, Nicole Bizzotto, Margareta Norberg

**Affiliations:** 1 Institute of Communication and Health Università della Svizzera italiana Lugano Switzerland; 2 Department of Psychology Umeå University Umeå Sweden; 3 Department of Epidemiology and Global Health Umeå University Umeå Sweden

**Keywords:** COVID-19, coronavirus, longitudinal studies, EMA, worry, fear, pandemics

## Abstract

**Background:**

The foray of COVID-19 around the globe has certainly instigated worries in many people, and lockdown measures may well have triggered more specific worries. Sweden, more than other countries, relied on voluntary measures to fight the pandemic. This provides a particularly interesting context to assess people’s reactions to the threat of the pandemic.

**Objective:**

The general aim of this study was to better understand the worried reactions to the virus and the associated lockdown measures. As there have been very few longitudinal studies in this area published to date, development of feelings of worry over time was analyzed over a longer range than in previous research. Affective variables, worry in particular, were included because most of the research in this field has focused on cognitive variables. To employ new methodology, ecological momentary assessment was used for data collection and a multilevel modeling approach was adopted for data analysis.

**Methods:**

Results were based on an unbalanced panel sample of 260 Swedish participants filling in 3226 interview questionnaires by smartphone over a 7-week period in 2020 during the rapid rise of cases in the early phase of the pandemic. Causal factors considered in this study included the perceived severity of an infection, susceptibility of a person to the threat posed by the virus, perceived efficacy of safeguarding measures, and assessment of government action against the spread of COVID-19. The effect of these factors on worries was traced in two analytical steps: the effects at the beginning of the study and the effect on the trend during the study.

**Results:**

The level of general worry related to COVID-19 was modest (mean 6.67, SD 2.54 on an 11-point Likert scale); the increase during the study period was small, but the interindividual variation of both the worry level and its increase over time was large. Findings confirmed that the hypothesized causal factors (severity of infection, susceptibility to the threat of the virus, efficacy of safeguarding, and assessment of government preventive action) did indeed affect the level of worry.

**Conclusions:**

The results confirmed earlier research in a very special case and demonstrated the usefulness of a different study design, which takes a longitudinal perspective, and a new type of data analysis borrowed from multilevel study design.

## Introduction

When a serious health threat approaches, people have to decide whether any protection measures are called for. When it comes to explaining or predicting self-protective behavior as a response to the threat of a communicable viral infection, theorizing and empirical results related to cognitive predictors are available in abundance. These are most prominently represented by the cognitive factors of threat severity [1] and perceived personal susceptibility [2] in the process of impression formation, the efficacy of measures against the threat (response efficacy [3]), and one’s ability to follow these measures (self-efficacy). Several conceptualizations and theories stand at hand to show a comprehensive picture of how all of these aspects fit together [4-10].

Besides these cognitive predictors, there are also elements of affective or emotional factors of self-protective behavior, which are treated together with cognitive factors in some cases and are treated separately in other cases. For instance, Harper and colleagues [11] summarize that research has paid substantial attention to public communication, but only little attention has been paid to character and emotion as factors of protection behavior. However, the literature on affective reactions in pandemics seems to be mainly concerned with the consequences rather than with the predictors of worry. In a study on the responses of a Chinese population to the pandemic, MacKay et al [12] reached a similar conclusion for the “routes to anxiety over disease contraction,” which they refer to as “understudied.” Assuming theoretical positions, Presti et al [13] argue that fear is an integral part of pandemics, which is mostly damaging but can also be contained (see also [14]). Early results from application of the Fear of COVID-19 Scale (FCV-192), a questionnaire designed for use worldwide, bear witness to this fact (eg, [15]).

Evidence of strong affective reactions to COVID-19 among the public is highlighted by the calls to action raised by Asmundson and Taylor [16,17], who refer to the first survey studies in connection with the global COVID-19 crisis. Several researchers have aimed to interpret the consequences of COVID-19 clinically and presented them as mental conditions [18,19], compared anxiety related to the present virus with the health anxiety trait measured several months earlier, and linked this anxiety with “cyberchondria,” an exaggerated need to seeking health information [20]. The concept of a behavioral immune system [12] was tested and largely confirmed, supporting the assumption that individuals take actions that involuntarily protect them from infection at times of a pandemic [21].

If fear indeed takes this role, this could explain the acceptance of restrictions in the fight against the virus that was found in a study from Denmark [22]. In a British study, Harper et al [11] assessed the self-perceived risk of contracting COVID-19, fear of the virus, moral rules, and political ideology on behavior change in response to the pandemic. Fear of COVID-19 turned out to be the only predictor of adopting protective measures [11]. In a Turkish study, the very same variable, fear of COVID-19, was shown to affect several mental variables [23]. These findings provide sufficient reason to perform more research on the potential effects of trait and situational emotional states, and in particular of worries and fears of communicable viral infections, on behavioral intentions for protecting oneself.

Worries and fear are not only important as a predictors of behavior but it is also relevant to ask how emotions develop and to identify their predictors. A longitudinally designed Chinese study of emotional reactions to fear of communicable diseases, severe acute respiratory syndrome (SARS) in this case, found that older and middle-aged people experienced less anger and had less need for emotion-focused coping skills in comparison with younger adults. Over the complete study period, emotion-focused coping increased more among the older and middle-aged population than among the younger participants; however, this trend was reversed at the peak of the SARS outbreak. The overall age differences were then reversed by the end of the outbreak. Findings of this study suggest that older adults may be better at emotional regulation than their younger counterparts: they react to a crisis with less anger and are better able to adapt their coping strategies to the changing environment [24]. This volatility of coping abilities motivated us to focus on worry, as a disagreeable and often uncontrollable state, in our present analysis. A review of demographic and attitudinal determinants of protective behaviors during a pandemic showed that being a woman, having a higher educational level, and being older are associated with behavior modification. Additionally, individuals’ perceived susceptibility to and severity of the disease, as well as stronger belief in the effectiveness of recommended safeguarding behaviors predict behavior change. Moreover, trust in authorities and a higher level of anxiety were also associated with compliance with protective behaviors [25].

Assuming that emotions affect behavior, identifying the causal influences on emotions is an eminent question. Focusing on three serious mental conditions as dependent variables (depression, anxiety disorders, and posttraumatic stress disorder), a study performed in Spain found that the elderly, the well-off, and people who felt adequately informed of COVID-19 developed these mental conditions less often, whereas women, people with a history of mental conditions, those with present COVID-19 symptomatology, and those with experience of others close to them having COVID-19 had increased mental health symptoms. Spiritual well-being was the best predictor for avoiding mental health symptoms and loneliness was the highest risk factor [18]. The measures taken against the spread of the pandemic also have to be considered as a separate source for adverse mental states [26].

There are few studies of the COVID-19 pandemic that assumed a longitudinal perspective. A German survey study showed that participants reported significantly increasing virus-related anxiety in the months before the survey. As these months were over when the survey was fielded, the information about the trend development had to be collected retrospectively. Cyberchondria in the pandemic was associated with current virus anxiety, and the association was moderated by the trait health anxiety. Subjectively adequate information on the virus lowered current virus anxiety [27].

A Chinese study of the mental health and affective consequences of confinement shortly after the virus appeared in Wuhan also adopted a longitudinal perspective by repeating measures from before confinement to 2 weeks into confinement. Such an approach may be adequate for studying the consequences of confinement on those who had to suffer from it, but does not suffice as a major step in assessing the time series on macrosocial responses to the pandemic [28]. As an aside, such research suggests, as do other experts in the field [13], that people should be put in quarantine only after benefits have been weighted against risks.

Based on these considerations, the aim of this study was to describe the development of Swedish people’s reaction to the spread of the COVID-19 virus (SARS-CoV-2), measured predominately as general worries related to the pandemic. The observations from previous studies highlighted above led to some conclusions for our study. As cognitive predictors of self-protective behavior have so far been studied more broadly than affective predictors, we were more interested in the latter, and finally chose a design that contained predictors of both types. The dynamic nature of affective reactions and fears demands studies with a longitudinal design; yet, such designs have hardly ever been employed in studies on the subject. To fill this gap, we used a longitudinal design with data from a range suitable to describe change over time during the very early phase of the COVID-19 pandemic. We also employed newly developed methods for data collection and data analysis. In a longitudinal design with high-frequency measures of 1 day, we employed an ecological momentary assessment (EMA) design for data collection. EMA has been discussed and successfully used for a variety of subjects [29-32]. For data analysis, a multilevel modeling approach was employed.

During the early phase of the COVID-19 pandemic, government assessment, communication, and actions taken highly differed between countries. Although many governments decided to lock down large parts of society in an attempt to curb the spread of the virus, the Swedish society, by contrast, was not closed, but safeguarding measures were launched, and the population was urged to voluntarily follow recommendations similar to a lockdown. Relying on voluntary recommendations gives the population’s perceptions and opinions of the threat created by the pandemic a special importance. At the same time, the expectations from the government and other authorities on individuals to show solidarity, take responsibility, and follow recommendations and regulations were strong in Sweden [33]. Some formal restrictions such as prohibition of visits to homes for the elderly and rules for distancing at restaurants were also implemented. During this period, the number of deaths increased dramatically and reached considerably higher levels compared to, for example, the surrounding Nordic countries. The Swedish government’s policy was widely debated and strongly questioned. For example, it was suggested in national and international media that the population was exposed to an “experiment.” However, by the end of April 2020, Sweden’s policy was also supported by the World Health Organization as a “role model” [34]. 

The aim of this study was to assess worries of COVID-19, predictors of these worries, as well as individuals’ evaluations of the lockdown measures in Sweden. Moreover, as there have so far been very few longitudinal studies in this research area, the aim was to study development over time for a longer range than adopted in previous research. Special attention was paid to affective reactions, the stronger of which are potentially harmful such as panic or maybe fear, and the weaker of which might support administrative measures that restrict liberties for a period of time.

## Methods

### EMA Design

The aim of this study was predominately descriptive as it intended to document levels and changes in worry at the individual level during the early phase of the COVID-19 pandemic in Sweden. The analysis also pursued a secondary aim to demonstrate that data collected by a smartphone with an EMA tool in daily rhythm can be a basis for meaningful analyses of the formation and change of emotions toward a phenomenon such as the COVID-19 pandemic. EMA was developed to study mood management and has typically been applied by paper and pencil before the ubiquity of the smartphone [35-38]. Respondents are contacted by EMA, with contacts serving as a reminder that a questionnaire is due, as a device to place the time of the interview at a particular hour of the day, or in juxtaposition to particular events. A meta-analysis turned to positive perceptions of one’s well-being and documented studies that asked participants questions up to 12 times a day that were to be answered on their phones [36]. Objective measures of physiological variables are compatible with the methodology, but have not been employed very often to date.

Huckins et al [38] took the chance to add another wave of interviews to an ongoing study of students’ mental health to assess the reactions toward the pandemic in the spring of 2020, employing smartphone EMA technology. They found students to be more depressed and more anxious than they had been in a comparison period prior to the pandemic. Our aim was to demonstrate the suitability of smartphone-based EMA technology beyond the study of mood.

### Recruitment and Sample

Initially, students at the Department of Psychology, Umeå University, Sweden, were informed of the study via email. An article describing the study in the local newspaper in Umeå also attracted participants. PRO, an organization for retired people in Umeå, was also approached. An online invitation letter was available from March 25 to May 17, 2020. A link to the website with the invitation letter was distributed across social media all over Sweden, and participants came from the whole country. These procedures clarify that we never aimed at a representative sample, and thus we do not claim representativity.

The invitation letter detailed the purpose of the study, use of the Smartphone Ecological Momentary Assessment (SEMA^3^) app [39] for distributing questionnaires, the load of questionnaires to be answered (how many, how often, how long), and informed of participants’ voluntary cooperation and rights; General Data Protection Regulation (GDPR) legislation was followed. Participants received an email for downloading the app. Participants first received an introduction survey covering sociodemographic variables, followed by daily surveys with thematic questions.

Owing to the different dates of entry to and exit from the study, different lengths of participation in the study, and different spacing between surveys, our sample presents unbalanced panel data [40]. Overall, 328 adults participated in the survey; given that three survey waves are considered to be the minimum for performing a multilevel analysis [41], we dropped all participants who only completed the first introductory survey and one or two additional surveys (n=68). 31% (21/68) completed only one survey, 31.5% (22/68) completed two, and 37% (25/68) completed three surveys, including the introduction survey. For inclusion in the study, participants had to (1) be of adult age (18 years or above), (2) be fluent in Swedish, and (3) have access to a smartphone (or a tablet).

The mean age of the analytical sample was 49.6 (SD 15.75) years, 76.7% (201/262) were women, and 80.9% (212/262) had received an academic education or were enrolled in such an institution at the time of the survey.

No gender, age, and education differences were found between dropouts and remaining participants, the latter of whom were included in the final sample (N=262). Participants in the final sample handed in between 4 and 28 surveys, including the introduction survey. This amounted to a mean of 12.3 (SD 5.46) surveys per participant. Table 1 presents an overview of how many participants replied to how many surveys. The total number of surveys we received from the sample of respondents was 3226. The analyses reported below are based on these cases.

**Table 1:** Number of questionnaires filled in per participant (N=268).

Number of completed interviews	Participants, n (%)
4	18 (6.7)
5	14 (5.2)
6	11 (4.1)
7	21 (7.8)
8	12 (4.5)
9	23 (8.6)
10	10 (3.7)
11	10 (3.7)
12	10 (3.7)
13	32 (11.9)
14	10 (3.7)
15	18 (6.7)
16	17 (6.3)
17	18 (6.7)
18	8 (3.0)
19	11 (5.2)
20	4 (1.5)
21	10 (3.7)
22	3 (1.1)
23	5 (1.9)
24	1 (0.4)
25	1 (0.4)
26	0 (0)
27	0 (0)
28	1 (0.4)

### Data Collection

We used the SEMA^3^ tool [39], a readily downloadable app at no charge to participants who possessed a smartphone with either the Android or iOS operating system. The tool was developed by a group of researchers at the Melbourne School of Psychological Sciences and is suitable for conducting intensive longitudinal survey research [42]. This tool allows for delivering surveys at fixed points in time or at fixed time intervals. During the period of data collection of almost 8 weeks, new questions were added to closely monitor the development, ask questions of relevance for participants, and assess reactions close in time to experiences, as in line with the EMA methodology.

Questions related to our dependent variable (general worry of COVID-19) and major independent variables (severity, susceptibility, and efficacy of safeguard measures) were asked every day. Other questions were scheduled at different intervals, covering aspects such as propensity for behavior change, personal response efficacy, and social factors such as loneliness due to the pandemic. Each day at 10 AM, a survey was released and participants then had 12 hours to complete it. The first thematic survey could appear on the same day as the introduction survey. Not all participants responded on a daily basis.

After approximately 14 days, participants were thanked, irrespective of the number of surveys they had handed in. They were also asked about their experience of taking part in the study and invited to continue at a lower rate, twice a week. Nevertheless, the dependent and major independent variables were contained in every questionnaire. Assessment of governmental actions appeared in the survey for the first time on April 1, 2020. Questions on specific aspects of worry were added to the survey from April 16, 2020, onward.

No personal data were collected since each participant was assigned a code without any link to the participant’s ID number or postal address. Moreover, no sensitive information was collected. A risk and vulnerability analysis was carried out in collaboration with the Information Technology Service Department at Umeå University according to a standardized protocol documenting information types and assessment of the information based on security aspects of confidentiality, accuracy, and accessibility. Thereafter, a risk analysis was performed.

### Measures

#### Survey Design

Most survey questions were adapted from previously used measures in a small pilot study, translated from English to Swedish, and tailored to fit the study setting. Questions on specific worries and trust in government were originally formulated in Swedish. Unless otherwise noted, the following measures were assessed on a 7-point Likert scale ranging from 1 (“do not agree”) to 7 (“agree”).

#### Worry

Worry was measured by a single item question, “To what extent are you worried about the coronavirus?” on a scale from 0-10, where 0 corresponds to “not worried at all” and 10 corresponds to “very worried” (mean 6.67, SD 2.54). This question was part of every survey, including the introduction survey. Using a single item is justified by the need to keep the daily questionnaire as short as possible to reduce the number of possible dropouts.

Specific worries were assessed with respect to five items once a week as of April 16: “Are you worried about getting infected by the coronavirus yourself?” “Are you worried about someone close to you being infected by the coronavirus?” “Are you worried that your personal finances have or will be affected by the spread of the coronavirus?” “Are you worried that the Swedish economy has or will be affected by the spread of the coronavirus?” and “Are you worried that the world economy has or will be affected by the spread of the coronavirus?” (specific worries combined: mean 3.98, SD 1.84 on a scale of 1-7; α=.71). The general worry item and the combined five specific worries showed good internal consistency (Cronbach α=.76). This indicates that the single-item measure produced results of quality comparable to the scaled 5-item measure and supports the validity of the single-item measure.

#### Perceived Severity of COVID-19

Severity of the disease was measured by three direct questions: “Corona is a threat to everyone”; “Fighting the coronavirus is not a matter of illness or health, it is a matter of life and death”; and “There is no greater health threat than Corona right now.” All three items were averaged to create a compound score (mean 5.29, SD 1.55; α=.84).

#### Perceived Susceptibility to COVID-19

Susceptibility was measured with three scaled questions about the risk of catching a disease: “Compared to others in my age group, I am less likely to be infected”; “I don’t think my family will get infected”; and “Even when the coronavirus gets closer, I don’t think I’ll get it.” Agreement with the statements was originally coded high, but as agreement signals low susceptibility, the three questions were reversed, with 1=low susceptibility and 7=high susceptibility. All three items were averaged to create a compound score (mean 2.51, SD 1.44; α=.84).

#### Efficacy of Safeguard Measures

The three items related to safeguard measures, “The actions taken so far can slow the spread of the coronavirus,” “The recommendations that apply to everyday behavior will work and will reduce the spread of the coronavirus,” and “Politicians responsible for public health will be able to control the spread of the coronavirus,” were combined, and a compound score was created (mean 2.51, SD 1.44; α=.91).

#### Assessment of Government

A single-item question was used to measure how people assessed governmental performance in management of the COVID-19 pandemic: “How do you assess the government’s way of handling COVID-19?” This was measured on a 10-point scale, ranging from 1 “The government makes the right decisions” to 10 “The government makes the wrong decisions” (mean 4.16, SD 2.97). This question appeared in the main survey as well as in the follow-up survey.

### Data Analysis

Analysis of the longitudinal data of being worried and other related factors was performed within a multilevel modeling approach [41]. The multilevel model for change allows investigating both change within and between individuals. The analysis of the within-person change (Level 1) concerns the individual development that each subject experiences over time and is attributable to a personal combination of different influence factors, whereas the change between subjects (Level 2) is related to influence factors that are common to groups of subjects in a given sample. In this study, we were particularly interested in the question of how the changes in worry differ between subjects according to different levels of perceived severity, perceived susceptibility, efficacy of safeguard measures, and the assessment of government. The two-level hierarchical models were estimated using a maximum-likelihood method in SPSS Statistics 25. The first step of the analysis involved the estimate of an unconditional mean model, which was followed by the unconditional growth model and other models in turn, each adding a new predictor. This procedure enables determining the relative contribution of each new variable on top of the factors that were already considered in the earlier models. More specifically, after having tested the unconditional mean (Model A) and unconditional growth (Model B) models, we added perceived severity (Model C), perceived susceptibility (Model D), efficacy of safeguarding (Model E), and finally agreement with government (Model F) to the model. All of these variables were tested as possible influence factors of both initial status and change: the intercept represents each subject’s average level of worry, while the coefficient on time indicates the increase based on each additional wave the participant took part in.

## Results

Descriptive statistics indicated that, on average, adults in our Swedish sample showed a level of worry of 6.20 at the very beginning of their participation in the study, which increased over time by an estimated 0.07 per day. Of note, large standard deviations were associated with both mean values, indicating that people differ widely with respect to their initial status of worry as well as with respect to their rate of change. The negative correlation coefficient between initial status and rate of change suggests that those with higher levels of worry at the beginning increased their worry level less rapidly compared with those who were initially less worried.

Table 2 presents an overview of all models that were tested. Model A represents the unconditional mean model, which provides information about the variation of the outcome worry. This model does not include either a time variable or any predictor. The mean value of worry across all occasions and individuals was 6.54 (on a scale from 0 to 10), indicating that the study participants were worried to some extent, between the two extreme values. The estimated within-person variance was 0.77, indicating that people do change their level of worry to some extent; between-person variance was 5.38, indicating a large amount of variation in worry among the individual participants of the study. The intercept as well as the two variance components were significant at the *P*<.001 level, meaning that adding additional variables may reduce the magnitude of the two variance components.

Model B, also called the unconditional growth model [41], includes the participation time of the individuals to the study as an additional predictor, enabling quantifying differences between participants with respect to the rate of change in their worries. According to Model B, the average change trajectory of participants had an intercept of 6.29 (*P*<.001) in worry and a slope of .045, significant at the *P*<.001 level, indicating that the level of worry increased between the end of March and the beginning of May. The within-person variance component (0.56, *P*<.001) of Model B summarizes the extent to which the data vary around the individual linear change trajectory (not around the person-specific mean), whereas the two variance components on level 2, initial status and rate of change, estimate the between-person variability in initial status (5.25, *P*<.001) and rates of change (0.015, *P*<.001). Adding other factors that would reduce the amount of variability in these components would help to improve the model fits. The fact that Model B is a better fit than Model A can be derived from a direct comparison, as shown by the values for *R^2^_e_* and *R^2^_0_*. The former represents the within-person residual in model A and B: comparing both values showed a decline of 27% (0.77–0.56/0.77=0.27), meaning that 27% of the variance is explained by introducing the time variable. The second value, *R^2^_0_*, represents the variance component at the outset. Comparing both models indicates an improvement of Model B with respect to this component by 2% (5.38–5.25/5.38=0.02). Therefore, including a time variable in the model particularly improved the estimate of worries at the outset.

The covariance component quantifies the association between the initial status of worry and its development over time. As such, this component helps to answer the question of whether people who are more worried at the beginning also become more (or less) worried over time. Reexpressing the covariance as a correlation coefficient [41], the relationship amounts to –0.16, meaning that those who were worrying more at the beginning are becoming slightly less worried over time. Overall, Model B showed that some of the within-person variation is associated with time, and that most of the variability in worry resided between the participants at the start, with only a small amount of variability, albeit significant, found in the change over time.

Model C and Model D bring in the aspects of threat appraisal. To facilitate interpretation, we centered perceived severity on its sample mean (5.044); to avoid giving individuals who participated in more waves greater weight in the model, mean centering was performed on the person-level data. Therefore, both intercepts, that of initial status (6.43) as well as that of the rate of change (0.03), represent the average fitted values, which were both significant at the *P<*.01 level. Participants with an average value of perceived severity showed a value of initial status that was 0.03 points higher. The estimated rate of change in worry for a participant with an average level of perceived severity of COVID-19 amounted to 0.01. Although this is a fairly small level of increase, it was significant at the *P*<.01 level, suggesting that during the study period, participants, on average, increased their level of worries about COVID-19.

**Table 2:** Models predicting general worry.

Parameter			Model A	Model B	Model C	Model D	Model E	Model F
**Fixed effects**								
	**Initial status**							
		Intercept	6.54*** (0.14)^a^	6.29*** (0.14)	6.43*** (0.14)	6.44*** (0.14)	6.43*** (0.13)	5.96*** (0.25)
		Severity	N/A^b^	N/A	.20*** (.04)	.21*** (.04)	.21*** (.04)	.19*** (.04)
		Susceptibility	N/A	N/A	N/A	.14*** (.04)	.13** (.04)	.14*** (.04)
		Efficacy of safeguarding	N/A	N/A	N/A	N/A	–.15** (.04)	–.11*** (.03)
		Agreement with government	N/A	N/A	N/A	N/A		.13** (.05)
	**Rate of change**							
		Intercept	N/A	.045*** (.01)	.03** (.01)	.03** (.01)	.03** (.01)	.04** (.02)
		Severity	N/A	N/A	.01** (.005)	.01* (.005)	.01* (.005)	.01** (.004)
		Susceptibility	N/A	N/A	N/A	–.01** (.004)	–.01* (.004)	–.01** (.004)
		Efficacy of safeguarding	N/A	N/A	N/A	N/A	.003 (.004)	–
		Agreement with government	N/A	N/A	N/A	N/A	N/A	–.002 (.003)
**Variance components**								
	Within-person (level 1)		0.77*** (0.02)	0.56*** (0.02)	0.52*** (0.02)	0.51*** (0.02)	0.51*** (0.02)	0.50*** (0.02)
	In initial status (level 2)		5.38*** (0.48)	5.25*** (0.48)	4.52*** (0.44)	4.48*** (0.44)	4.40*** (0.43)	4.36*** (0.45)
	In rate of change			0.015*** (0.002)	0.013*** (0.002)	0.013*** (0.002)	0.013*** (0.002)	0.011** (0.002)
	Covariance			–0.045* (0.02)	–0.065** (0.02)	–0.06** (0.02)	–0.06** (0.02)	–0.06** (0.02)
	*R^2^_e_*			0.27	0.071	0.02	0	0.01
	*R^2^_0_*			0.02	0.14	0.01	0.02	0.04
	*R^2^_1_*			N/A	0.13	0	0	0.15
Deviance			9441	8869	7265	7238	7218	6649
AIC^c^			9447	8881	7281	7258	7242	6675
BIC^d^			9466	8918	7328	7317	7312	6750

^a^Numbers in parentheses denote the standard error.

^b^N/A: not applicable (not included in the model).

^c^AIC: Akaike information criterion.

^d^BIC: Bayesian information criterion.

*P<.05, **P<.01, ***P<.001.

Considering the variance components of Model C, we found that the within-person variance decreased from 0.56 to 0.52, which corresponds to a small reduction by 0.7%. The reduction of variance in the initial status was more remarkable at 14%, from 5.25 to 4.52, by adding severity as a predictor that explains levels of worry at the beginning of the participants’ trajectory. Given that this value is significantly different from 0 (*P*<.001), other factors may be added to the model to explain the existing variance in Model C. Additionally, the variance component of rate of change, *R^2^_1_*, diminished by 13% (from 0.15 to 0.13) by introducing the predictor of perceived severity. Given that *R^2^_1_* remained significantly different from 0, other predictors may still reduce the amount of the variance in this component of Model C.

In Model D, we added susceptibility, the other component of threat appraisal, which should further explain why people increase their worries over time. As in the previous analysis, we used the mean-centered value of susceptibility (5.401). This addition brought forth the following conclusions. First, controlling for the effects of susceptibility on initial status and rate of change, the effects of severity on initial status and rates of change on participants’ worries amounted to .21 (*P*<.001) and .01 (*P*<.05), respectively. Second, keeping the value of severity constant, the effects of susceptibility on initial status and rates of change on participants’ worry amounted to .14 (*P*<.001) and –.01 (*P*<.01), respectively, meaning that participants who differed by one point on perceived susceptibility at the initial status showed higher levels of worry by .14. Even if they were more worried at the beginning, their average rate of change was .01 lower, indicating that participants who believed they were more susceptible at the beginning revealed a slower rate of increase of worry over time compared with those who felt less worried at the initial status; in other words, susceptibility was negatively associated with the rate of change in worry.

When we added susceptibility as a predictor of initial status of worry as well as of the rate of change, the amount of variance also shrank to some extent. The within-person variance was reduced from 0.52 to 0.51 (*P*<.001), the initial state variance dropped by approximately 1% point from 4.52 to 4.48 (*P*<.001), and the rate of change variance remained unchanged.

To improve the model further, we added perceived e*fficacy of the safeguard* *measures.* Again, we mean-centered the variable (perceived efficacy mean 5.3) to facilitate interpretation of the coefficients. Considering that the addition of perceived efficacy of safeguarding in Model E indicates an effect on levels of worry in the expected direction, holding perceived severity and susceptibility constant, two people who differ by 1 point in their view of whether safeguards were effective or not did show a difference in the level of worry by –.15. In other words, the less people were convinced that safeguard measures were effective, the more were they worried about COVID-19. Although this effect was significant at *P<*.001, no effect was found with respect to the rate of change (.479), meaning that people did not change their minds about safeguards measures. In the following model, we therefore dropped the perceived efficacies of safeguard measures as a predictor of the trajectory, but not as a predictor of initial status. Given the impact of attributed efficacy of safeguard measures on the initial status of worry, the corresponding variance component in Model E shrank from 4.48 to 4.40, representing a decrease of almost 2%.

The final model, Model F, added the new predictor of to what extent people think that the government properly handled the COVID-19 crisis in Sweden. Assessment of government predicted the initial status of worry but not the change of worry over time: keeping all other variables (perceived severity, susceptibility, and efficacy of safeguarding measures) constant, lower levels of agreement with government measures (expressed by the low end of a scale of 1-10) indicated a higher level of being worried about the virus (β=.13, *P*=.008). Given that assessment of government performance did not change the trajectory of worry over time, we finally excluded this variable as a predictor of rate of change.

The deviance statistics, including the Bayesian information criterion and Akaike information criterion, indicate how the models improve by adding the single variables. Additionally, as recommended by Singer and Willett [41], the pseudo-R statistic was computed for the within-person variance, the initial status, as well as for the rate of change variance components to show how the variance components decreased from model to model, which indicates a growing quality of the model.

## Discussion

### Principal Findings

Foremost, this analysis demonstrates the suitability, and perhaps even the necessity, of this type of statistical model building. Further, according to our formulated aims with regard to EMA methodology, this study also shows that meaningful data collection can be achieved by employing EMA along with using smartphones to collect data. There are several aspects to consider in interpreting the model-building results.

Research to date tells us that the self-perceived susceptibility to fall victim to a threat, the perceived severity of a risk (ie, the damage it can do), the belief in the efficacy of institutional safeguarding measures taken, and trust in government or other institutions responsible for public health are among the causal factors of risk assessment and related variables. The first noteworthy general result of our study is that the particular case of the COVID-19 pandemic confirms previous results with a very special context, a different study design (ie, a longitudinal perspective), and a new type of data analysis as a crucial methodological innovation. The predictors mentioned have a strong cognitive component. In contrast, worries and their development over time belong to the factors with a strong affective component. How the two are linked is not yet clear and needs to be addressed in future research.

This innovation carries potential to enlarge the analytical perspective. When we also look at the data taking temporal development into account, we found an impact of more or less the same variables as those that emerged in previous cross-sectional studies. The impact was actually found for all causal variables when the distribution of the worries at the beginning of the study period was predicted. This similarity is the second general result.

However, it was not only the worries in the beginning alone that were affected but there were also effects on the trends in the development of worries. These effects represent the third important general result, which clearly suggests that a cross-sectional analytic design would have missed an important part of the reality of people’s thinking about COVID-19 and the dynamic nature of the predictors. The fact that not all predictors produced linear trends shows that differentiation is called for, which we highlight as the fourth main finding. In our case, the contribution of perceived efficacy of the safeguarding measures and the support for the government did not have an impact on the trajectory of worry, and the impact on the initial status of worry must be considered to be modest. This might be surprising due to the rapid increase in COVID-19 mortality rates in Sweden and the intense debate regarding the Swedish policy. However, this might be linked to the largely unchanged policy in Sweden during the period under study, as well as a generally high and stable trust among Swedes in the public health agency, the health care system, other societal authorities and institutions as well as in the government [43]. Another reason for the small effects on worry trends is that very few participants stayed on board and handed in information over the entire study period of approximately 8 weeks. The average number of surveys handed in was 12.3. Therefore, even if significant effects were shown, the possibility to demonstrate larger shifts in the trend was small.

As stated above, effects on initial status appear to be stronger and more stable than effects on rate of change. The effect on initial status is the totality of everything that happened between the start of consciousness about the pandemic and a person’s entry into the study. Mass communication effects have long been known to be particularly strong in the early phases of an issue. The earliest phase was not included in our analysis. However, the influences in this pre-early phase may have overshadowed later effects.

Worries are clearly affected by perceptions of the threat, which is determined by scientific observations. Mustering the defensive forces to decide how to protect people from a health threat might leave more worries than the situation demands. The finding that agreement with government emerged as the weakest predictor might also have to do with the high trust in authorities and government in Sweden, especially during the period of data collection in this study [43].

Among the interesting observations from this study was the high level of spread in the worry measure. This can, as a matter of speculation, be considered a consequence of the participants being introduced into the study continuously almost over the complete data collection period. Those recruited early replied in a situation when there were still very few COVID-19 cases in the country. In addition, the question about assessment of government had a different point of reference at different times as some regulations were altered. The comparatively small change over time might have been due to a rather large share of participants taking part for quite a short period of time only. However, the daily questions might have mitigated the level of worry. We cannot exclude some automatic response behavior among participants in our study. Taking time for reflecting on our questions during a limited time each day, and in a format that was easy to handle, might have decreased the participants’ worry for the rest of the day. 

The classic factors of protection motivation, severity, and susceptibility considered in our study may have been overshadowed by other variables such as demographic and attitudinal variables [25] and response efficacy (ie, the belief in the benefits of one’s own and the government’s measures) [44,45].

The long Swedish tradition of political stability and trust in government may have contributed, along with a broad consensus among the political parties, at least early in the pandemic, to an impression that the fight against the virus was in good hands.

### Limitations

A study with so many measurement points in time has to rely on modern digital technology, which makes it somewhat of a challenge to control sampling. For practical purposes, it might be best not even to try to control access to the questionnaire and to filing the responses. This means that a sample for such a study can take the form of a panel, with repeated application of the questionnaire to the same people; however, this will result in erratic schedules of participation and high differences in the number of days a person filed answers.

Since three interviews are generally considered the minimum for a multilevel analysis, and these three were collected within 2 days in some cases, there was not much time available to witness change. In fact, this is not a disadvantage as the pace of change is an open research issue, which could be advanced with data such as those collected in this study at times of a pandemic threat, given that individuals report during longer time periods. Moreover, it is a limitation that our analytical sample cannot be claimed to be representative of the source population. It is also a limitation that we could not ask about residency for anonymity reasons. This means we cannot link the responses on perceptions to a local or regional condition, in particular the number of people infected. This should be considered when interpreting the results.

Other limitations were technical problems with the SEMA app, such as difficulties in downloading the app and that participants did not receive any survey in some instances, or the response disappeared if the participant received a phone call at the same time. The result was that many were interested but could not participate and that some information might be lost. We had no information about participants’ COVID-19 infection status, and therefore no conclusions can be drawn about the impact of an infection on the level of worry.

Limitations also originate from data that do not quite meet the standards to be applied to survey research when used to produce a final word on a controversial issue, in which all insecurity in the meaning of a study had better be avoided. Representative samples and validated measures produce results that are easier to trust than those obtained with our study design. Nevertheless, our research question does not live up to standards suitable for more final research.

## Abbreviations

EMA: ecological momentary assessment
FCV-192: Fear of COVID-19 Scale
GDPR: General Data Protection Regulation
SARS: severe acute respiratory syndrome
SEMA: Smartphone Ecological Momentary Assessment app
